# Differences in Immune Response and Biochemical Parameters of Mice Fed by Kefir Milk and *Lacticaseibacillus paracasei* Isolated from the Kefir Grains

**DOI:** 10.3390/microorganisms9040831

**Published:** 2021-04-14

**Authors:** Viera Karaffová, Dagmar Mudroňová, Marián Mad’ar, Gabriela Hrčková, Dominika Faixová, Soňa Gancarčíková, Zuzana Ševčíková, Radomíra Nemcová

**Affiliations:** 1 Department of Morphological Disciplines, University of Veterinary Medicine and Pharmacy, Komenského 73, 04181 Košice, Slovakia; viera.karaffova@uvlf.sk (V.K.); zuzana.sevcikova@uvlf.sk (Z.Š.); 2 Department of Microbiology and Immunology, University of Veterinary Medicine and Pharmacy, Komenského 73, 04181 Košice, Slovakia; marian.madar@uvlf.sk (M.M.); dominika.faixova@student.uvlf.sk (D.F.); sona.gancarcikova@uvlf.sk (S.G.); radomira.nemcova@uvlf.sk (R.N.); 3 Institute of Parasitology, Slovak Academy of Sciences, Hlinkova 3, 04001 Košice, Slovakia; hrcka@saske.sk

**Keywords:** multi-strain, single-strain, lactic acid, immunity

## Abstract

The health benefits of kefir consumption have been well-known for hundreds of years. The objective of this study was to investigate the effect of kefir milk and the probiotic strain *Lacticaseibacillus paracasei* Ž2 isolated from kefir grains on the immune response and selected parameters of the lipid and liver enzymatic profiles of mice. Mice fed with kefir milk showed significantly increased phagocytic activity and percentages of B cells in the blood and increased gene expression for mucins and percentages of CD8+ lymphocytes in the gut. By applying kefir, we achieved a significant reduction in serum LDL cholesterol and an LDL/HDL ratio that favored an increase in HDL cholesterol. Regarding the hepatic enzymes, in particular a significant reduction in ALT activity was observed. *L. paracasei* Ž2 alone stimulated the immune response more markedly compared with kefir milk. Regarding the systemic level, we observed increases in the proportion of all T cells (CD3^+^), CD4^+^ lymphocytes and the ratio of CD4+:CD8+ cells, and regarding the local intestinal level we noted a significant increase in gene expression for mucins (MUC-1 and MUC-2) and IgA. Moreover, we confirmed the formation of a biofilm on the surface of the forestomach only after the application of *L. paracasei* Ž2 alone, but not after kefir administration. The results confirmed the hypothesis that the final effect of the probiotic does not correspond with the effect of the individual strain but is the result of mutual interactions of the microorganisms presented in a preparation, and therefore in the case of multi-strain probiotics, in vivo testing of the complex preparation is necessary.

## 1. Introduction

Kefir is an acid-alcoholic fermented dairy product of low acidity and creamy consistency, produced in the Balkans, Eastern Europe and the Caucasus. It can be produced by fermenting milk with commercial lyophilized starter cultures of kefir, traditional kefir grains and the product that remains after the removal of kefir grains [1]. *Lacticaseibacillus paracasei* subsp. *paracasei* (previously *Lactobacillus paracasei*), *Lactobacillus acidophilus*, *Lactobacillus delbrueckii* subsp. *bulgaricus*, *Lactiplantibacillus plantarum* (previously *Lactobacillus plantarum*) and *Lactobacillus kefiranofaciens* are among the species predominant in kefir grains. More than 23 different types of yeasts were isolated from kefir grains. The predominant species include *Saccharomyces cerevisiae, Saccharomyces unisporus, Candida kefyr* and *Kluyveromyces marxianus* subsp. *Marxianus* [2].

Regular consumption of kefir is associated with improved lactose digestion and tolerance; antibacterial, hypocholesterolemic and anti-hypertensive effects; plasma glucose control; and antioxidant, anti-carcinogenic and anti-allergic activity [3,4,5]. There are many studies supporting these findings but most of them have been performed on in vitro or animal models. Therefore, further systematic clinical trials are needed to better understand the effects of the regular use of kefir as part of a diet and their effectiveness in disease prevention [6].

*L. paracasei* is a gram-positive, facultative heterofermentative species of lactic acid bacteria (LAB). It is a part of the normal intestinal micro-biota, in which it influences Th1/Th2 equilibrium through increased production of Th1 cytokines, activity of macrophages and production of antibodies [7]. On the other hand, it also reduces the production of pro-inflammatory cytokines in human intestinal dendritic cells infected with salmonellae [8]. Hill et al. [9] reported that *L. paracasei* and products fermented by it are capable of alleviate allergies, preventing damage of the gastric mucosa and inhibiting the accumulation of adipose tissue. Overall, it seems that *L. paracasei* modulates inflammation differently based on different conditions in a host organism.

Contradictory results have been obtained in the studies comparing the efficacy of single-strain and multi-strain probiotics in inhibiting pathogens or other medical indications [10,11,12,13]. However, less is known about the effectiveness of multi-strain probiotics, in terms of whether the mixing of strains results in synergistic effects on the host immune system or whether the efficacy is decreased due to mutual inhibition of the present strains.

In agreement with the previous statement, the aim of this study was to investigate the effect of kefir milk and the probiotic strain *Lacticaseibacillus paracasei* Ž2 isolated from kefir grains on the selected parameters of the immune response, lipid metabolism and liver enzymatic profiles of mice.

## 2. Materials and Methods

### 2.1. Preparation of Bacterial Inocula

Kefir milk was prepared according to the manufacturer’s instructions from 15 g of washed Tibetan kefir grains (Milchema s.r.o, Považská Bystrica, Slovakia) containing 26 strains of lactic acid bacteria (*L. acidophilus*, *L. cellobiosus*, *L. parakefir* sp. nov., *L. fructivorans*, *L. kefiri*, *L. plantarum*, *L. paracasei* subsp. *paracasei*, *L. hilgardii*, *L. casei* subsp. *rhamnosus*, *L. brevis*, *L. delbrueckii* subsp. *bulgaricus*, *L. lactis*, *L. helveticus* subsp. *lactis*, *L. kefiranofaciens*, *L. plantarum*, *L. delbrueckii* subsp. *lactis*, *L. kefir granum* sp. nov., *L. casei* subsp. *pseudoplantarum*, *Lactococcus lactis* subsp. *lactis*, *L. lactis* var. *diacetylactis*, *L. lactis* subsp. *moris*, *Streptococcus salivarius* subsp. *thermophilus*, *S. lactis*, *Enterococcus durans*, *Leuconostoc cremoris*, *Lc. mesenteroides*), 2 strains of acetic acid bacteria (*Acetobacter aceti*, *A. rasens*) and 14 strains of yeasts (*Candida kefir*, *C. pseudotropikalis*, *C. rancens*, *C. tenuis*, *Kluyveromyces lactis*, *K. marxianus* var. *marxianus*, *K. bulgaricus*, *K. fragilis*, *Saccharomyces lactis*, *S. carlsbergensis*, *S. unisporus*, *Debaryomyces hansenii*, *Torulopsis holmii*, *Zygosaccharomyces rouxii*). The grains were placed in 250 mL of sterile milk (Milk Agro s.r.o., Prešov, Slovakia) and were fermented for 24 h at room temperature. Subsequently, the grains were removed from the kefir milk by straining.

*Lacticaseibacillus paracasei* Ž2 (previously *Lactobacillus paracasei* Ž2) was isolated from Tibetan kefir grains (Milchema s.r.o., Slovakia) at the Department of Microbiology and Immunology, University of Veterinary Medicine and Pharmacy, in Košice, Slovakia. The strain showed promising probiotic properties—strong inhibitory activity against bacterial pathogens (*S. aureus*, *S.* Typhimurium, *Escherichia coli* O149:F4, *Bacillus cereus*), high resistance to gastrointestinal conditions (gastric juice, bile salts), formation of biofilm, no harmful enzymatic or hemolytic activity and susceptibility to antibiotics required by the EFSA (European Food Safety Authority) Commission in 2012 [5]. The strain is registered in GenBank under number MT682913. *L. paracasei* Ž2 was cultured overnight on MRS (DeMen-Rogosa-Sharpe) agar (Carl Roth GmbH, Karlsruhe, Germany) at 33 °C under anaerobic conditions (BBL GasPak Plus™, Franklin Lakes, BD, USA). The bacterial colonies were harvested and diluted to an optical density of 1 Mc Farland in phosphate-buffered saline (MP Biomedicals, Illkirch-Graffenstaden, France).

### 2.2. Design of the Experiment

The experiment was realized at the Institute of Parasitology of the Slovak Academy of Sciences in Košice, Slovakia. The Ethical Committee of the University of Veterinary Medicine and Pharmacy in Košice and the State Veterinary and Food Administration of the Slovak Republic approved the experimental protocol number 318/14-221, and the animals were handled and sacrificed in a humane manner. Eighteen six-week-old female BALB/c mice (Velaz, Prague, Czech Republic) were divided into 3 groups of 6 animals each. Mice were reared in a controlled atmosphere with free access to water and were fed ad libitum with feed composed of 92.77% dry matter, 27% nitrogen substances, 8.60% fat, 1.70% fiber, 1.83% Ca, 0.18% Mg, 0.27% Na, 0.75% K, 1.60% K (Peter Miško, s.r.o., Snina, Slovakia).

Groups of mice were treated for 11 days and daily received100 μL of freshly prepared kefir milk in the KEF group, 100 μL of freshly prepared *L. paracasei* Ž2 inoculum in the LP group (the concentration was adjusted to OD 1 McFarland corresponding to approx. 5 × 10^8^ cfu/mL) and 100 μL of PBS in the control group, using an intragastric feeding needle. On day 12, all mice were anesthetized using sodium pentobarbital (86 mg/kg). Blood samples were collected by means of retro-orbital blood collection and then animals were euthanized by cervical dislocation. Intestines and mesenteric lymph nodes were immediately removed from the sacrificed mice and used for further analysis.

### 2.3. Phagocytic Activity Analysis

Phagocytic activity was analyzed in peripheral blood using Phagotest^®^ (ORPEGEN Pharma, Heidelberg, Germany) according to the manufacturer’s guidelines. Briefly, precooled heparinized fresh whole blood (50 µL) was mixed with 10 µL of FITC-labeled opsonized *E. coli* (2 × 10^9^ bacteria/mL). From each sample 2 tubes—test and control—were prepared. The test tube was incubated for 10 min at 37 °C in a water-shaking bath and the control tube stayed on ice. Immediately after incubation, the quenching solution (50 µL) and subsequently 1.5 mL of wash solution were added to each sample. The tubes were centrifugated (5 min, 250× *g*) and supernatant was discarded. The washing was repeated once again with 1.5 mL of wash solution. Blood cells in sediments were lysed and fixed with 1 mL of lysing solution for 20 min at laboratory temperature. After subsequent centrifugation (5 min, 250× *g*), the sediments were washed with 1.5 mL of wash solution. Finally, 100 µL of DNA staining solution was added and samples were incubated for 10 min on ice before analysis. For a flow cytometric analysis, a six-color BD FACSCanto^TM^ flow cytometer equipped with the blue (488 nm) and red (633 nm) lasers (Becton Dickinson Biosciences, San Jose, CA, USA) was used. Data were analyzed using BD FACS Diva^TM^ software.

### 2.4. Homogenization of Tissues and Isolation of Total RNA of IgA, MUC-1 and MUC-2

Tissue samples (jejunum, ileum and cecum) were cut into 20-mg pieces, immediately placed into RNA Later solution (Qiagen, Manchester, UK) and stored at −70 °C prior to RNA purification. A single tissue fragment was transferred into 1 mL of TRI Reagent (Molecular Research Center, Cincinnati, OH, USA), and homogenized using 2.7 mm of zirconium silica beads (BioSpec Products, Bartlesville, OK, USA) in a Magnalyser (Roche, Indianapolis, IN, USA). To separate the phases, 50 μL of 4-bromanisole (Molecular Research Center, USA) was added. The whole content of the tube was centrifuged and the upper aqueous phase was collected for total RNA purification using the RNAeasy mini kit (Qiagen, UK) following the manufacturer’s instructions. An Ambion^®^ Turbo DNA-free kit (Life Technologies, Carlsbad, CA, USA) was used for the treatment of RNA samples to remove genomic DNA. Both the purity and concentration of RNA were detected spectrophotometrically on a NanoDrop 200c (Thermo Scientific, Madison, WI, USA) and 1 μg of the total RNA was immediately reverse transcribed using the iScript cDNA Synthesis Kit (Bio-Rad, Hercules, CA, USA). The resulting cDNA was 10× diluted in nuclease-free water (Qiagen, UK) and used as a template in a quantitative real-time PCR.

### 2.5. Relative Expression of IgA, MUC-1 and MUC-2 by Quantitative Real-Time PCR

The mRNA level of IgA MUC-1 and MUC-2 were determined. In addition, mRNA relative expression of the reference gene, coding GAPDH (glyceraldehyde-3-phosphate dehydrogenase), was determined and used for data normalization. The primer sequences used for qRT-PCR are listed in Table 1. All primer sets allowed DNA amplification efficiencies between 94% and 100%.

Amplification and detection of specific products were performed using the CFX 96 RT system (Bio-Rad, USA). Subsequent qRT-PCR to detect expression of the mouse immune genes was performed for 35 cycles at the annealing temperature for each pair of primers (Table 1). A melting curve from 50 °C to 95 °C with a reading every 0.5 °C was performed for each individual qRT-PCR plate. Each sample was subjected to real-time PCR in duplicate and mean values of duplicates were used for subsequent analysis. We also confirmed that the efficiency of amplification of each target gene (including GAPDH) was essentially 100% in the exponential phase of the reaction, in which the quantification cycle (Cq) was calculated. The Cq values of the selected genes were normalized to an average Cq value of the reference gene (ΔCq), and the relative expression of each gene was calculated as 2^–ΔCq^. These expression levels were then used for comparative data analysis.

### 2.6. Isolation of Lymphocytes from Mesenteric Lymph Nodes

Lymph nodes were placed in ice-cold Hanks balanced salt solution–HBSS (137 mM NaCl, 5 mM KCl, 1.1 mM Na_2_HPO_4_·2H_2_O, 0.4 mM KH_2_PO_4_, 5 mM D-glucose, 4 mM NaHCO_3_, 10 mM HEPES, pH 7.1–7.3, filtered 0.22 µM). The nodes were pulped between two sterile glass slides and the obtained material was mixed with 5 mL of HBSS. The suspension was filtered through a 70-µm nylon cell strainer (BD Falcon, Glendale, CA, USA) into a 15-mL centrifuge tube. Tubes were centrifuged at 250× *g* for 5 min. The sediment was washed twice with HBSS by centrifugation at 250× *g* for 5 min and then counted after staining with Türk solution in a Bürker chamber and adjusted at 1.10^6^ cells per 50 μL.

### 2.7. Lymphocyte Identification

Identification of lymphocyte subpopulations in peripheral blood and mesenteric lymph nodes was performed using flow cytometry. Fifty microliters of heparinized blood or cell suspension from lymph nodes was incubated with monoclonal antibodies for 15 min in the dark at room temperature. Lymphocytes were identified using two combinations of antibodies—CD4/CD8a and CD3/B220—the detailed specifications of which are given in Table 2. After incubation, 500 μL of lysis solution (BD FACS Lysing Solution, BD Biosciences, San Jose, CA, USA) was added to the tubes and then the tubes were incubated for 15 min in the dark at room temperature and the contents of the tubes were washed twice with 1 mL PBS (MP Biomedicals, Illkirch-Graffenstaden, France). PBS solution (100 μL) was added to the tubes before measurement. The analyses were performed on the above-described flow cytometer. Proportions of lymphocytes are expressed as a percentage.

### 2.8. Serum Biochemical Analysis

Serum samples obtained from individual animals were used for the determination of several basic biochemical parameters, in order to assess the effect of kefir and *L. paracasei* Ž2 administration on clinical status. An automated Ellipse (AMS, Rome, Italy) biochemical analyser and standard kits (Dialab, Prague, Czech Republic) were used to determine concentrations of the following biochemical parameters: total protein (TP); albumin; triacylglycerols (TG); HDL-cholesterol; LDL-cholesterol; activities of enzymes aspartate aminotransferase (AST), alanine aminotransferase (ALT) and alkaline phosphatase (ALP).

### 2.9. Histological Processing of Stomach and Histo-Fluorescent In-Situ Hybridisation Analysis

For detection of lactobacilli in biofilms on the mucosal surface of the gastrointestinal tract (GIT), the tissue samples were embedded in paraffin, then the sections were prepared and a Histo-FISH method standardized in our laboratory by Madar et al. was applied [18]. In short, the stomachs with parts of the cardia were extracted from the gastrointestinal tract of mice (*n* = 6), washed in PBS (pH 7.2) without removing the inner content and fixed immediately in Carnoy’s solution for 4 h at 4 °C in a ratio of 6:3:1 (100% ethanol; chloroform; glacial acetic acid). After fixation in Carnoy’s solution, the samples were dehydrated through a series of ethanol concentrations (100%, 96%, 90%, 70%) embedded in paraffin blocks and subjected to the classical histological processing. Paraffin sections (10-µm thick) were mounted on glass slides (SuperFrost Plus, Braunschweig, Germany). Sections prepared from different parts of tissue blocks were selected based on their proximity to the forestomach and were stored in a dark box at 4 °C for further processing. In the next step, selected slides were deparaffinised by xylen and gently rehydrated in a graded alcohol series from 90% to 50%, followed by washing in PBS for 5 min. This step is necessary to prevent the disruption of the formed biofilm. The best quality slides were selected for subsequent processing by means of the FISH protocol. First, slides were coated with an enzymatic mixture according to Czerwinski et al. [19]. Subsequently, the enzymatic mixture was removed and slides were gently washed 3 times with PBS.

The hybridization buffer, prepared according to Czerwinski et al. [19] was filtered, heated to 52 °C and then mixed with probe Lab158 in a ratio of 50 µL:2 µL (buffer:probe). For the detection of *Lactobacillus* spp. probe Lab158 5’GGTATTAGCAYCTGTTTCCA, labeled with 6-carboxyfluorescein on the 5′ end (6-FAM) with excitation 470 nm and emission 525–550 nm (green colour), was used. The probe was synthesized and HPLC-purified by Sigma Aldrich (St. Louis, MO, USA). The hybridization of sections was carried out overnight at 52 °C in a dark hybridization humid chamber. For the visualization of other bacteria and tissue cells present on histological sections, the slides after termination of hybridization were stained with 4′,6-diamidino-2-phenylindole (DAPI) dye (excitation at 365 nm and emission at 445–450 nm) for 10 min, washed in PBS, then were immersed in Vectashield mounting medium and covered by coverslips to prevent photobleaching (Vector Laboratories, Burlingame, CA, USA). The tissue sections were visualized by means of epifluorescence microscopy with a Carl Zeiss Axio Observer Z1 microscope and images were analyzed with AxioVision Rel.4.6 software (Carl Zeiss, Oberkochen, Germany).

The viability of microorganisms in the inoculum prior to application to mice was tested using the Viability Fluorescent Quick Test on Polycarbonate Filter (VFQTOPF), in which live bacteria stained with carboxy-flurescein diacetate (CFDA) are seen as green and dead bacteria stained with propidium iodide (PI) are seen as red [20].

### 2.10. Statistical Analysis

Statistical differences between groups were determined using the Tukey’s post-test after one-way analysis of variance (ANOVA). Statistical analysis was performed in the GraphPad Prism version 3.00 statistical program. Values are expressed as medians ± standard deviation (SD) for gene expression results and means ± standard deviation (SD) for other parameters.

## 3. Results

### 3.1. Phagocytic Activity

**Figure 1:**
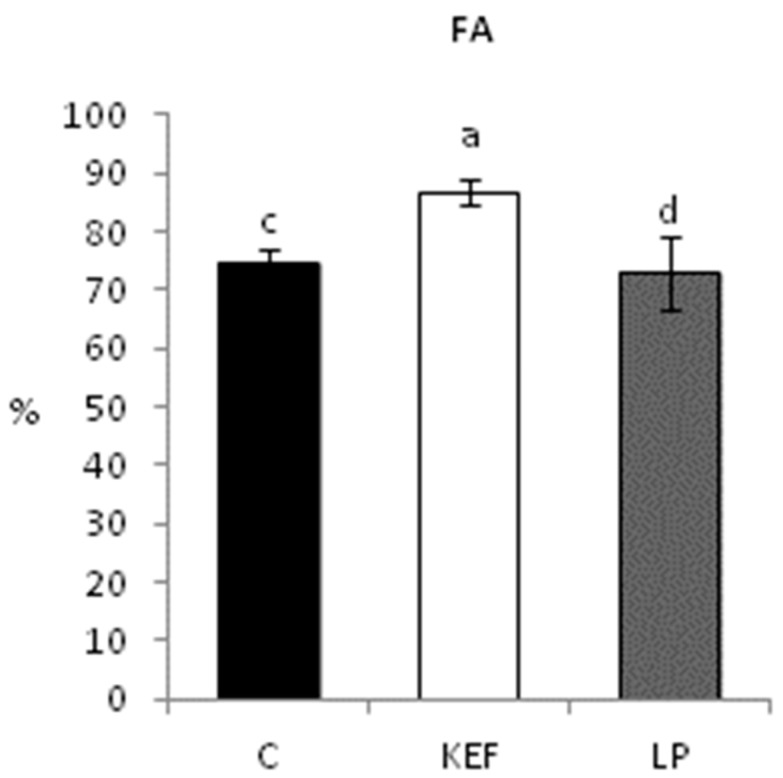
Effect of application of *L. paracasei* Ž2 and kefir on phagocytic activity in the peripheral blood of mice (*n* = 6). ^ab^ *p* < 0.05; ^ac^ *p* < 0.01; ^ad^ *p* < 0.001.

The phagocytic activity (Figure 1) was significantly higher in the kefir group compared to both the control and LP groups. There was no difference in the level of phagocytic activity between the control and LP groups. The ability of phagocytes to engulf the fluorescently labeled *E. coli* (MFI = mean fluorescence per 1 phagocyte) was not significantly affected.

### 3.2. Relative Expression of MUC-1, MUC-2 and IgA, Evaluated by Quantitative Real-Time PCR

**Figure 2:**
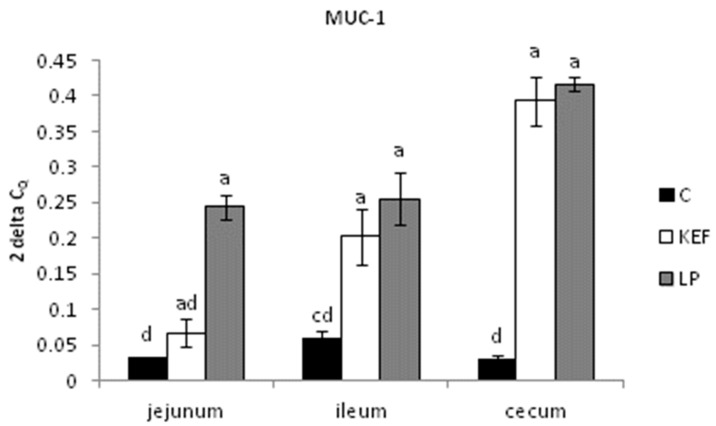
Relative expression level of MUC-1 (jejunum, ileum, cecum) in mice (*n* = 6). Results at each timepoint are the median of 2^–ΔCq^. Means with different superscripts in individual intestinal segments are significantly different. ^ab^ *p* < 0.05; ^ac^ *p* < 0.01; ^ad^ *p* < 0.001.

The relative expression of MUC-1 in the jejunum was upregulated in the LP group compared to the other groups (*p* < 0.001). Similarly, a trend was observed for gene expression in other parts of intestine (ileum, cecum) in the LP group compared to the control (*p* < 0.001) (Figure 2).

**Figure 3:**
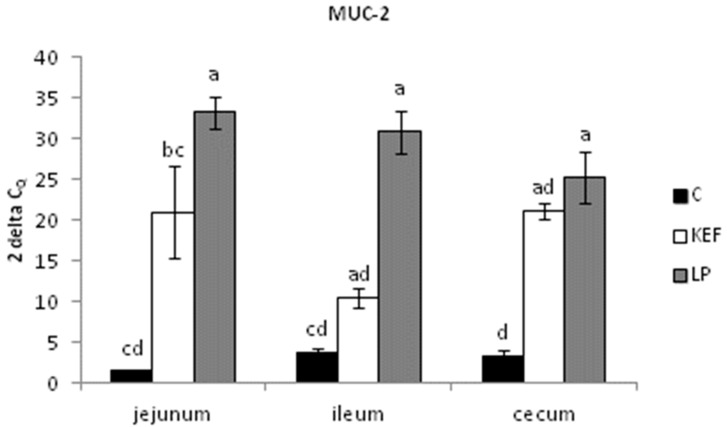
Relative expression level of MUC-2 (jejunum, ileum, cecum) in mice (*n* = 6). Results at each timepoint are the median of 2^–ΔCq^. Means with different superscripts in individual intestinal segments are significantly different. ^ab^ *p* < 0.05; ^ac^ *p* < 0.01; ^ad^ *p* < 0.001.

The relative expression of MUC-2 in the jejunum and cecum was markedly upregulated in the LP group compared to the control (*p* < 0.001) and KEF group (*p* < 0.05). The same tendency was recorded for MUC-2 gene expression of the ileum in the LP group compared to other groups (*p* < 0.001) (Figure 3).

**Figure 4:**
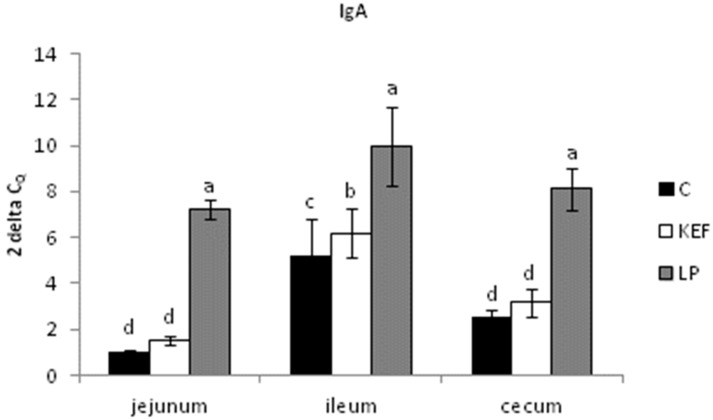
Relative expression level of IgA (jejunum, ileum, cecum) in mice (*n* = 6). Results at each timepoint are the median of 2^–ΔCq^. Means with different superscripts in individual intestinal segments are significantly different. ^ab^ *p* < 0.05; ^ac^ *p* < 0.01; ^ad^ *p* < 0.001.

The relative expression of IgA in the jejunum and cecum was upregulated in the LP group compared to the other groups (*p* < 0.001). Similarly, IgA gene expression in the ileum was significantly upregulated in the same group compared to the control (*p* < 0.01) and KEF group (*p* < 0.05) (Figure 4).

### 3.3. Proportions of Lymphocyte Subpopulations in Peripheral Blood

**Figure 5:**
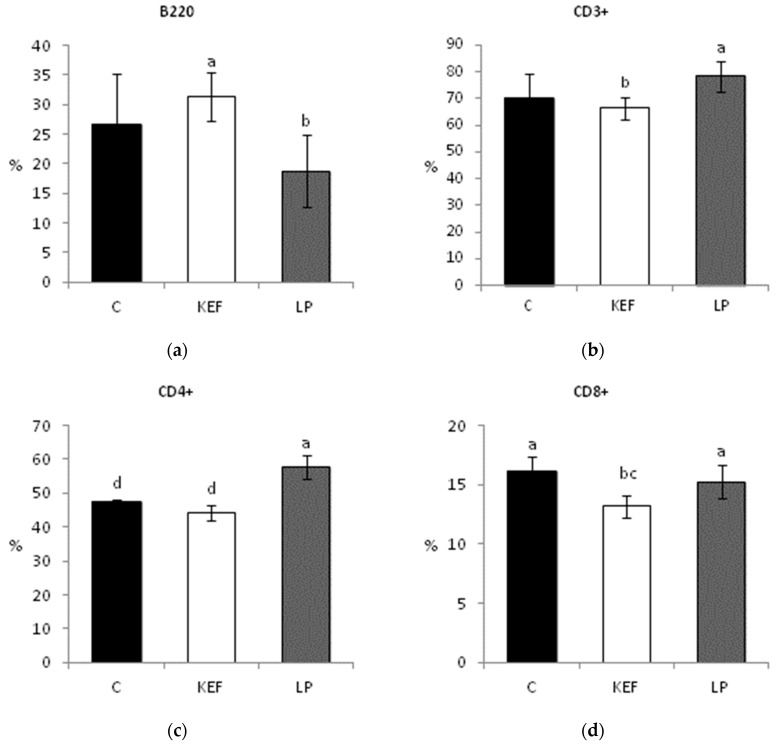

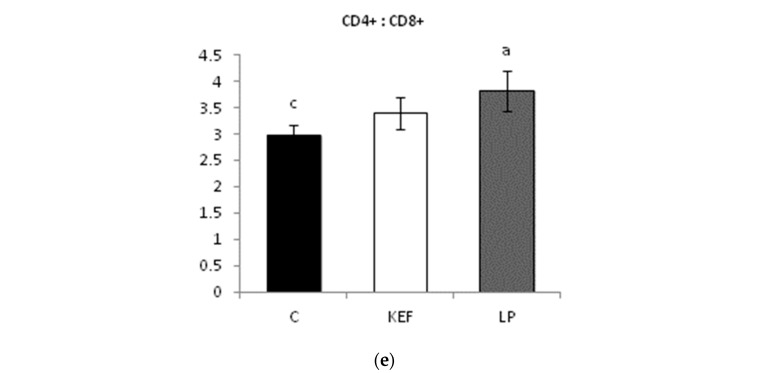
Effect of application of *L. paracasei* Ž2 and kefir on percentages of: (**a**) B220, (**b**) CD3^+^, (**c**) CD4^+^ and (**d**) CD8^+^ and (**e**) ratio of CD4^+^:CD8^+^ lymphocytes isolated from peripheral blood in mice (*n* = 6). ^ab^ *p* < 0.05; ^ac^ *p* < 0.01; ^ad^ *p* < 0.001.

The total percentage of lymphocytes after the application of the kefir isolate LP and kefir milk was not significantly affected. The proportion of all lymphocytes in the control group (56.1% ± 2.8%) was only slightly higher in comparison with the kefir group (53.6% ± 2.8%) and LP group (53.0% ± 7.0%). In the kefir group, the percentage of B lymphocytes (B220^+^) was significantly higher compared to the LP group (Figure 5a) and, conversely, the percentage of T lymphocytes (CD3^+^) was lower (Figure 5b). The opposite trend was observed in the *Lactobacillus* group, in which a slight increase in T lymphocytes and, conversely, a decrease in B lymphocytes (although with no statistically significant differences) were recorded compared to the control group.

The highest proportion of the helper T lymphocytes (CD4^+^) was recorded in the LP group, in which their percentage was significantly higher compared to the control and kefir groups (Figure 5c). The proportion of cytotoxic T lymphocytes (CD8^+^) was significantly lower in the kefir group compared to the control and LP groups (Figure 5d). The ratio of CD4^+^:CD8^+^ lymphocyte, the increase of which is an indicator of immuno-stimulation, was significantly higher in the LP group compared to the control group (Figure 5e). There were no statistically significant differences in the percentage of CD4^+^CD8^+^ double-positive lymphocytes (data not shown).

### 3.4. Proportions of Lymphocyte Subpopulations in Mesenteric Lymph Nodes

The proportion of lymphocyte subpopulations in mesenteric lymph nodes was only minimally affected by the application of the kefir isolate *L. paracasei* Ž2 and kefir milk (data not shown). There were no significant changes in the total number of lymphocytes, T lymphocytes or T helper or double-positive lymphocytes. We noted non-significantly higher proportions of B lymphocytes (B220^+^) in both experimental groups as compared to the control. The only significant difference was noted in the proportion of cytotoxic CD8^+^ lymphocytes, which were significantly increased in the kefir group in comparison with LP and control groups (in both cases *p* < 0.05).

### 3.5. Nitrogen Profile

**Figure 6:**
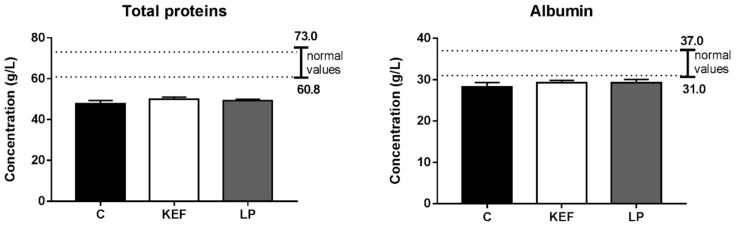
Effect of application of *L. paracasei* Ž2 and kefir on the concentration of total proteins and albumin in the serum of mice following treatment. The ranges of recommended normal values are indicated by lines. No significant differences were detected among individual groups.

The nitrogen profile in the control group was affected by hypoproteinemia, which, however, was not accompanied by a significant decrease in albumin and total proteins, probably as a result of reduced exogenous protein supply, rather than reduced synthesis or loss in animals. Eleven-day application of kefir milk and inoculum with *L. paracasei* Ž2 had no significant effect on improving the nitrogen profile, and despite the moderately elevated serum levels of total proteins and albumin in both groups (KEF, LP), hypoproteinemia persisted (Figure 6).

### 3.6. Enzymatic Profile

**Figure 7:**
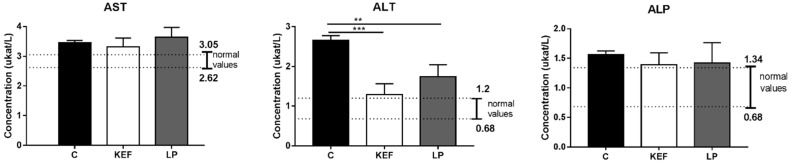
Effect of application of *L. paracasei* Ž2 and kefir on activities of serum enzymes alanine aminotransferase (ALT), aspartate aminotransferase (AST) and alkaline phosphatase (ALP) in mice (*n* = 6). The ranges of recommended normal values are indicated by lines. ** *p* < 0.01; *** *p* < 0.001.

The highest activities of the liver-specific enzyme ALT were recorded in animals of the control group (Figure 7). Although the increase in this activity did not exceed three times the reference value, confirming severe hepatic impairment, its 2.2-fold increase indicates mild hepatic tissue irritation, probably due to increased energy intake in feed. Application of kefir and strain *L. paracasei* Ž2 had a positive effect, resulting in the significant reduction of ALT activities (*p* ˂ 0.001 and *p* ˂ 0.01, resp.) in KEF and LP groups of animals compared to the control. The decline in ALT activity, especially in the KEF group, to its physiological interface, indicates the initiation of the repair processes of hepatic tissue. At the same time, the beneficial effect of kefir milk application on the reduction, albeit insignificant, of non-specific liver enzymes AST and ALP was confirmed (Figure 7).

### 3.7. Lipid Profile

**Figure 8:**
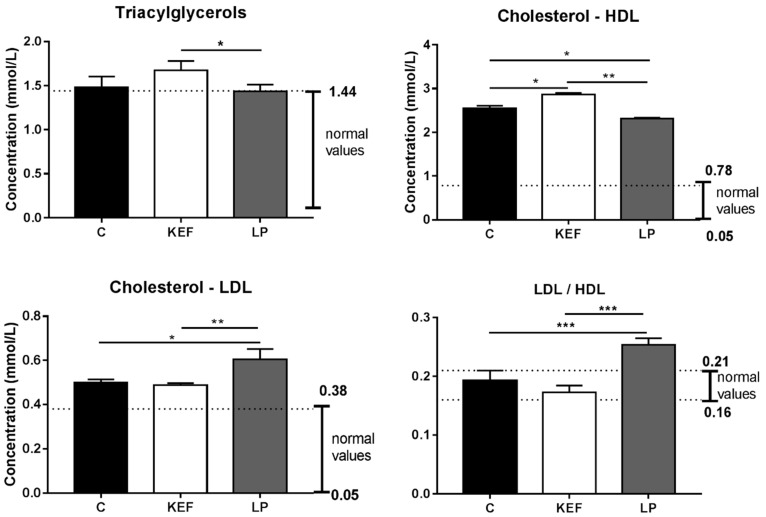
Effect of application of *L. paracasei* Ž2 and kefir on the concentration of serum triacylglycerols (TG), HDL cholesterol, LDL cholesterol LDL and LDL/HDL ratio in mice (*n* = 6). The ranges of recommended normal values are indicated by lines. * *p* < 0.05; ** *p* < 0.01;*** *p* < 0.001.

We recorded serum concentrations above physiological levels of triacylglycerols as well as of HDL and LDL cholesterol in the control group (Figure 8), indicating an elevated energy intake from feed, confirming a higher fat content than the recommended maximum of 5%. When assessing the selective parameters of the lipid profile, higher levels of cholesterol, HDL and LDL cholesterol are less informative than the LDL/HDL ratio. In the control group, this value was within reference levels, indicating that sufficient metabolic processes for fat breakdown occurred in the liver. The relatively higher fat intake in the KEF group was characterized by a significantly higher concentration of TG (*p* < 0.05) compared to the LP group and a significantly higher level of HDL-cholesterol (*p* < 0.05, *p* < 0.01) compared to both groups (C, LP). The application of kefir milk showed a hypocholesterolemic effect, confirmed not only by a significantly lower level of LDL-cholesterol (*p* < 0.01), but also by a significantly lower LDL/HDL ratio (*p* < 0.001) compared to the LP group. Application of *L. paracasei* Ž2 alone did not significantly affect fat loss and significantly higher levels of mainly LDL-cholesterol (*p* < 0.05, *p* < 0.01) or LDL/HDL cholesterol ratio (*p* < 0.001) compared to C and KEF, indicating a higher burden on hepatic tissue. This finding is also confirmed by the slightly higher release of AST and ALT enzymes (Figure 7) as compared to KEF, but only at the level of irritation and reversibility. Despite the effect on lipid metabolism, no significant effect on the body weight of the mice was observed at the time of autopsy, when the mice weighed 19.7 ± 0.26 g in the control group, 20.02 ± 0.23 in the LP group and 20.05 ± 0.21 g in the KEF group.

### 3.8. Biofilm Formation by Lactobacilli in the GIT

**Figure 9:**
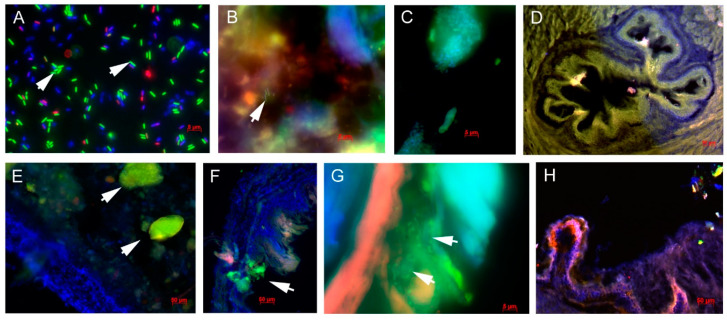
Results of Histo-FISH analysis. (**A**)—Viability fluorescent quick test on polycarbonate filter (VFQTOPF) showing viability of microorganisms in the inoculum containing *Lacticaseibacillus paracasei* Ž2; arrows indicate live bacteria (cFDA) depicted in green, whereas dead cells (PI) are depicted in red, and blue represents the DNA of cells stained with DAPI. For the detection of *Lactobacillus* spp. a Lab158 prove was used, labeled with 6-FAM emitting green fluorescence. DNA of cells was stained with DAPI, emitting blue fluorescence (**B**–**H**). (**B**)—Detection of *Lactobacillus* spp. (arrow) in the stomach content of the LP group. (**C**)—Kefir grain with *Lactobacillus* spp. bacteria (green) in the stomach content of the KEF group. (**D**)—Histological section of the gastric cardia with minimal biofilm formation (LP group). (**E**,**F**)—Particles composed of mostly *Lactobacillus* spp. (arrows) detected close to the forestomach surface in the KEF group. (**G**)—Biofilm formed by *Lactobacillus* spp. on the mucin layer on the forestomach surface in the LP group. (**H**)—No occurrence of *Lactobacillus* spp. on the forestomach surface of control mice.

Bacterial viability in the inoculum of *L. paracasei* Ž as well as kefir reached about 75% (Figure 9A). The high viability of bacteria in the inoculum is a prerequisite for successful colonization or biofilm formation in the GIT. We detected *Lactobacillus* spp. in the gastric content of both LP (Figure 9B) as well as kefir (Figure 9C) groups. In the kefir group, lactobacilli were mostly bound to kefir grains. In the cardia part of the stomach, we did not record biofilm formation in either the LP or the KEF group (Figure 9D). The highest incidence of *Lactobacillus* spp. were noted on the surface of the forestomach. In the KEF group, *Lactobacillus* spp. occurred mainly in the form of clumps on the mucosal surface (Figure 9E,F), whereas in the LP group lactobacilli formed a biofilm on the surface of the mucin layer (Figure 9G). No *Lactobacillus* spp. bacteria were observed in the control group (Figure 9H).

## 4. Discussion

Kefir culturing, originating from the Caucasus Mountains and also from Tibet, is over 5000 years old. Russian doctors began researching kefir in the 19th century and published scientific studies on its beneficial effects on human health. The extremely old age of the Caucasian population has been attributed to the regular consumption of kefir milk and fresh water [21]. It has been reported by Vinderola et al. [22] that kefir modulates the immune response in mice and increases the number of IgA+ cells in the intestinal and bronchial mucosa and the phagocytic activity of peritoneal and pulmonary macrophages. Similarly, in our study, the phagocytic activity was significantly increased after the application of kefir milk, but it was not influenced by the administration of *L. paracasei*. Llewellyn and Foey [23] summarized the knowledge about the influence of probiotic bacteria on phagocytic activity, including cell signaling. Their review provides a number of references to studies that have confirmed an effect on the activity of phagocytes at the local intestinal level, as well as at the systemic level. The link between local intestinal stimulation by probiotic bacteria and the systemic immune response is documented by their influence on cytokine production, including chemokines, pattern recognition receptors and signaling molecules. The migration of activated phagocytes from the intestine to other parts of the body, such as the spleen and blood, is also expected.

At the Department of Microbiology and Immunology of the University of Veterinary Medicine and Pharmacy in Košice, we isolated several *Lactobacillus* isolates from kefir grains, which were subsequently characterized and tested for probiotic properties. *L. paracasei* Ž2 showed a high resistance to simulated gastric juice and bile salts, biofilm formation and strong inhibitory activity against potential bacterial pathogens. Moreover, the strain was susceptible to all antibiotics required by EFSA and showed no harmful enzymatic or hemolytic activity [5]. Interestingly, the strain did not stimulate phagocytic activity, which is typical for many probiotic bacteria.

The beneficial effect of lactic acid bacteria is very closely related to the natural function of the intestinal microbiota, in that it modulates (among other processes) the composition of microbiota and stimulates the immune system. In addition, lactobacilli are able to support the intestinal barrier by means of an increase in tight junction proteins and the number of goblet and Paneth cells [24].

Certain *Lactobacillus* species have been shown to increase mucin expression in the intestine [25,26]. Our results also demonstrate the stimulatory effect of *L. paracasei* Ž2 alone on mucin gene expression, suggesting that this strain induces goblet and epithelial cells to secrete mucins, reducing the binding of enteric pathogens to the mucosal surface. It should also be noted that after gastrointestinal passage, *L. paracasei* strains are able to increase their adherence to mucins and epithelial cells in vitro*,* a factor that is important for maintaining the strain itself in the intestinal environment to exert its probiotic action [27]. Likewise, *L. acidophilus* A4 increased the MUC-2 gene expression in HT29 intestinal epithelial cells, and even this effect was not dependent on adhesion to the cell [28]. It was also observed that a probiotic formula, VSL#3 (*Bifidobacterium* spp., *Lactobacillus* spp., *Streptococcus thermophilus*), improved intestinal barrier function through upregulation of MUC-2 gene expression in mucin-deficient mice [29]. Ouwehand et al. [30] observed that a multi-strain probiotic composed of *L. reuteri, L. rhamnosus* and *P. freudenreichii* or *L. reuteri* alone did not affect mucin production in the colons of the elderly. In our experiment, the administration of kefir milk as a multi-strain probiotic did not significantly influence mucin gene expression in comparison with *L. paracasei* Ž2 alone. In spite of this, the addition of kefir increased mucin gene expression mainly in the cecum and ileum as compared to the control.

In another study, Carasi et al. [31] demonstrated an increase in IgA production along with inducing mucin, decreased expression of pro-inflammatory mediators and increased anti-inflammatory molecules following administration of *L. kefiri* CIDCA 8348 in healthy mice. Several authors observed that LAB significantly induces IgA secretion in the intestine [32,33,34] through an increase in the number of IgA^+^ B cells in mice [35]. This is consistent with our current results, in which *L. paracasei* Ž2 alone significantly increased the level of IgA gene expression in all parts of the intestine. In the intestine, an important role of mucosal IgA is the neutralization of harmful bacteria and viruses by influencing their ability to adhere to epithelial cells and their motility [36]. Similarly, Arai et al. [33] found that orally administered *L. paracasei* MCC1849 itself induced antigen-specific IgA production and a rise in the proportion of IgA^+^ B cells in the intestine, serum and lungs of mice.

In our experiment, we did not observe statistically significant differences in the percentages of all lymphocytes. Nurliyani et al. [37] administered goat milk fermented with kefir and goat milk to which a *Lactobacillus acidophilus* starter culture was added to rats and they subsequently monitored hematological parameters. They found that kefir did not affect the total lymphocyte count but *L. acidophilus* slightly reduced the total lymphocyte count, thus achieving similar results as those of our experiments.

The proportion of B lymphocytes in the peripheral blood in the kefir group was significantly higher compared to the LP group, where, on the contrary, the percentage of T lymphocytes was significantly higher. It seems that *L. paracasei* Ž2 stimulates more T lymphocytes on the systemic level, which was confirmed by the higher percentage of CD4+ cells, as well as the highest CD4^+^:CD8^+^ ratio as an indicator of immuno-stimulation. We expected to onserve a stronger effect of probiotics on local intestinal cellular immunity, which was not confirmed in our study, and surprisingly, the effects of kefir as well as *L. paracasei* Ž2 alone on the systemic cellular immune response were weaker than expected. Kim et al. [38] fed mice with chemically induced atopic dermatitis with a multi-strain probiotic consisting of *L. acidophilus* CBT LA1, *L. plantarum* CBT LP3, *Bifidobacterium breve* CBT BR3 and *B. lactis* CBT BL3 and they studied systemic as well as intestinal immune responses. They found that administration of the probiotic mixture significantly increased the proportion of CD4^+^ T cells in Peyer’s patches but no changes were observed in mesenteric lymph nodes and the spleen. Their results also showed the induced differentiation of naïve T cells toward Th1 and Tregs, and thus inhibited the Th2 immune response. Similarly, kefir-originating *L. kefiranofaciens* M1 has been shown to stimulate the Th1 immune response and decrease the production of Th2 cytokines in allergic patients [39]. Adiloğlu et al. [40] also observed a shift in the immune response towards the Th1 type in healthy volunteers after kefir consumption. Immuno-stimulation, manifested by a higher proportion of CD4^+^ T cells and activated B cells in Peyer’s patches, was observed in mice fed with kefir and subsequently infected with *Giardia intestinalis*. The percentage of observed lymphocyte subpopulations in mesenteric lymph nodes was not significantly affected by kefir application. CD8^+^ lymphocytes were unaffected in either Peyer’s patches or mesenteric lymph nodes. These changes were accompanied by significantly higher numbers of IgA-positive cells and higher gene expression for IFN-γ and TNF-α in the small intestine [41]. On the other hand, treatment of peripheral blood mononuclear cells received from patients with diagnosed pulmonary tuberculosis with kefir led to the stimulation of the Th2 type of the immune response, with elevated production of IL-10 and no significant changes in the proportions of CD4^+^ and CD8^+^ lymphocytes [42]. These results indicate that the immune response after treatment with beneficial bacteria also depends on the clinical status of an organism. In the study performed on healthy BALB/c mice, the authors observed increased numbers of IL-4- and IL-6-, as well as IL-10-producing cells in small intestines, indicating the induction of both the Th1 and Th2 immune responses [43]. In our trial, kefir significantly increased the percentage of CD8^+^ T cytotoxic cells in the mesenteric lymph nodes, suggesting rather a local stimulation of Th1 cellular immunity. This finding is also supported by the very weakly influenced stimulation of IgA gene expression in the intestinal mucosa after the application of kefir. In contrast, *L. paracasei* Ž2 alone significantly stimulated the antibody IgA response at the local intestinal level and T cell immunity at the systemic level. As mentioned above, the results of studies comparing the effect of multi-strain and single-strain probiotics considerably differ, which is summarized in the detail in the work of Ouwehand et al. [10]. It is evident from our study that the interrelationships between microorganisms in multi-strain probiotics are essential for the final action of the preparation. The desired effect of the probiotic preparation depends on the purpose of its use. For example, when administered to allergic patients, a shift in the immune response in favor of the Th1 type is required, with a predominance of T-regulatory lymphocyte activation and production of anti-inflammatory cytokines such as IL-10 and TGF-β. However, such an effect of the preparation is undesirable when used in most infectious diseases or cancer. As discussed by Ouwehand et al. [10], the potential benefits associated with the use of multi-strain probiotics (e.g., a broader spectrum of efficacy or synergistic effect of probiotic strains), may not be met in practice and the results of comparative studies are often not clear and exact. However, despite the confirmation of a synergistic microbiological effect (e.g., the improvement of growth or colonization properties, a wider spectrum of pathogen antagonism), multi-strain probiotics should also be tested for their immunological effects on the organism, even if the efficacy of the used individual strains is known.

In addition to its nutritional composition and immunomodulatory action, kefir has beneficial effects on blood and liver lipid profiles [44]. It has also been shown to decrease serum and hepatic cholesterol and TG levels [45], suggesting that kefir may be associated with the metabolism of triacylglycerols, the major storage form of excess calories [46]. With the increased intake of nutritionally balanced feed (in case of ad libitum feeding), the opportunity is created for increased energy intake from feed, with the possibility of storing fats in the body, which is accompanied by the higher demands, especially on metabolism in the liver. In the present study, the control group showed mild hepatic tissue irritation, manifested by the increased release of liver biomarkers (AST, ALT, ALP) and higher serum concentrations of TG, HDL and LDL cholesterol. By applying kefir, we achieved a significant reduction in serum LDL cholesterol and an LDL/HDL ratio in favor of an increase in HDL cholesterol; however, the concentration of TG was the highest, which could be caused by application of kefir milk, containing additional fat. Regarding the hepatic enzymes, in particular a significant reduction in ALT activity was observed. These results are consistent with the conclusions of a study by Choi et al. [44], who, by applying the powdered form of kefir in diet-induced obese mice, achieved a reduction in the activity of serum enzymes ALT and AST, total cholesterol and LDL-cholesterol. The concomitant decrease in the expression of genes associated with adipogenesis and lipogenesis has supported the claim that kefir prevents liver steatosis and has significant potential in clinical applications in the prevention and treatment of non-alcoholic fatty liver disease (NAFLD) [21,47,48]. Several studies using individual bacteria isolated from kefir showed similar improvements in plasma or serum lipid profiles, but similarly to our study using *L. paracasei* Ž2, no significant changes in HDL-cholesterol levels were observed [21,49,50].

We also tested biofilm formation by *L. paracasei* Ž2 and kefir in the GIT of mice. Based on our previous studies, we assumed the strongest biofilm formation would occur in the mouse forestomach [18], which was also confirmed in this work. We confirmed the potential to form a biofilm in *L. paracasei* Ž2 strain under in vitro conditions [5]. As confirmed by Histo-FISH analysis, *L. paracasei* Ž2 also forms a biofilm in vivo, on the surface of the mucin layer of the forestomach. The mucin layer has been shown to play a very important role in biofilm formation. It seems that the strong biofilm formation in the forestomach of mice corresponds to a thicker layer of mucin and a more acidic environment, suitable for lactic acid bacteria, as compared to the cecum, which is characterized by a small amount of mucin and very diverse complex microbiota [18,51]. This is also consistent with our finding that the *L. paracasei* Ž2 strain increased gene expression for both MUC-1 and MUC-2. Interestingly, we did not observe biofilm formation after the application of kefir, only finding clusters of *Lactobacillus* spp. bacteria associated with the forestomach surface.

In conclusion, *L. paracasei* Ž2 isolated from kefir grains affected the immune response of mice more markedly than multi-strain kefir milk. Strain Ž2 alone stimulated local intestinal immunity by means of a significant increase in gene expression for mucins (MUC-1 and MUC-2) and IgA, as well as at the systemic level by activating the T cell response. Kefir milk significantly stimulated phagocytic activity, increased the proportion of B lymphocytes in the blood and CD8+ cytotoxic cells in the mesenteric lymph nodes, whereas the effect on mucins and IgA in the intestine was weaker compared to *L. paracasei* Ž2. On the other hand, kefir positively influenced lipid and hepatic metabolism, which is manifested in particular by reducing fat accumulation in the liver and through the reduction of serum cholesterol. It is apparent from the results that the final effect of the probiotic does not correspond to the effect of the individual strain, but is the result of mutual interactions of the microorganisms contained in the preparation, and therefore it is necessary to test a complex probiotic mixture. For these reasons, we hypothesize that the *L. paracasei* Ž2 strain alone should be used mainly for short-term stimulation of immunocompromised patients, whereas kefir is suitable for long-term use in terms of its positive effect on metabolic parameters.

## Figures and Tables

**Table 1:** List of primers used in qRT-PCR for IgA, MUC-1 and MUC-2 mRNA detection in mice.

Primer	Sequence 5′–3′	Annealing/Temperature Time	References
IgA For	TCTCCTCCTCTTCTTGTCATACGC	58 °C/40 s	[14]
IgA Rev	GGAGGTAAGTACCACAGGAGCGTTT		
MUC-1 For	CCACCACTCCAGTTTACAGT	56 °C/30 s	[15]
MUC-1 Rev	GAATGATAGCTGAGCCTGAC		
MUC-2 For	GCTGACGAGTGGTTGGTGAATG	60 °C/30 s	[16]
MUC-2 Rev	GATGAGGTGGCAGACAGGAGAC		
GAPDH For	CATCACTGCCACCCAGAAGACTGTGGA	60 °C/30 s	[17]
GAPDH Rev	TACTCCTTGGAGGCCATGTAGGCCATG		

**Table 2:** The specifications of the anti-mouse monoclonal antibodies (eBioscience, San Diego, CA, USA) and amounts used for 50 μL of blood or cell suspension from mesenteric lymph nodes.

Type	Fluorochrome	Clone	Isotype	Concentration	Amount
anti-CD4	FITC	GK 1.5	IgG2b, κ	0.5 mg/mL	2 μL
anti-CD8a	R-PE	53–6.7	IgG2a, κ	0.2 mg/mL	1 μL
anti-CD3	PerCP-eFluor710	17A2	IgG2b, κ	0.2 mg/mL	2 μL
anti-B220	APC	RA3–6B2	IgG2a, κ	0.2 mg/mL	1 μL

## Data Availability

The data presented in this study are available on request from the corresponding author.
